# Supplementary material for: Structural and biochemical basis of interdependent FANCI‐FANCD2 ubiquitination
Source: EMBO J. 2022 Nov 17;42(3):e111898. doi: 10.15252/embj.2022111898 (PMC9890228; doi:10.15252/embj.2022111898)
Supplement: Supplementary file 1 — Expanded View Figures PDF [file EMBJ-42-e111898-s003.pdf]

Expanded View Figures

Figure EV1. Cryo-EM analysis and structure modelling of I_{Ub}D2-DNA complex.

A Example micrograph with scale bar.
B Example 2D classes. Circular mask is 170 Å in diameter. 2D classes surrounded by a green box correspond to I_{Ub}D2-DNA complex particles, while smaller-sized 2D classes surrounded by a red box likely correspond to monomeric I_{Ub}/D2 proteins.
C Fourier Shell Correlation (FSC) curves: between the two half maps produced in the final local non-uniform refinement (shown in blue) and between the refined model and final map (shown in orange).
D Particle orientation (viewing direction distribution) in the final map. Total number of particles: 139,601.
E *Top*: Locally filtered map coloured by local resolution, viewed from three different angles. *Bottom*: Corresponding structural model viewed under same orientations.
F I_{Ub}D2-DNA structure with corresponding map density (locally filtered map), centred on the isopeptide bond between K523 of FANCI and G76 of ubiquitin. Some well-resolved side-chains are illustrated as sticks and indicated.
G Interaction between FANCI and FANCD2 C-termini with corresponding map density (locally filtered map). A beta-sheet consisting of a FANCI and a FANCD2 strand is formed (residues 1,285–1,289 of FANCI and residues 1,384–1,388 of FANCD2). This is held in place through hydrophobic and electrostatic interactions with a FANCD2 helix (1,351–1,377 aa). Residues predicted to participate in such interactions are shown as sticks and indicated. Selected side chains, for which there is good density are also shown as sticks. For clarity, adjacent to that region elements of the I_{Ub}D2-DNA structure and map are not shown.
H I_{Ub}D2-DNA structure centred on DNA. Density corresponding to the 27 bp modelled DNA is shown as orange mesh. Colouring of structure is as in (E–G).

© 2022 The Authors

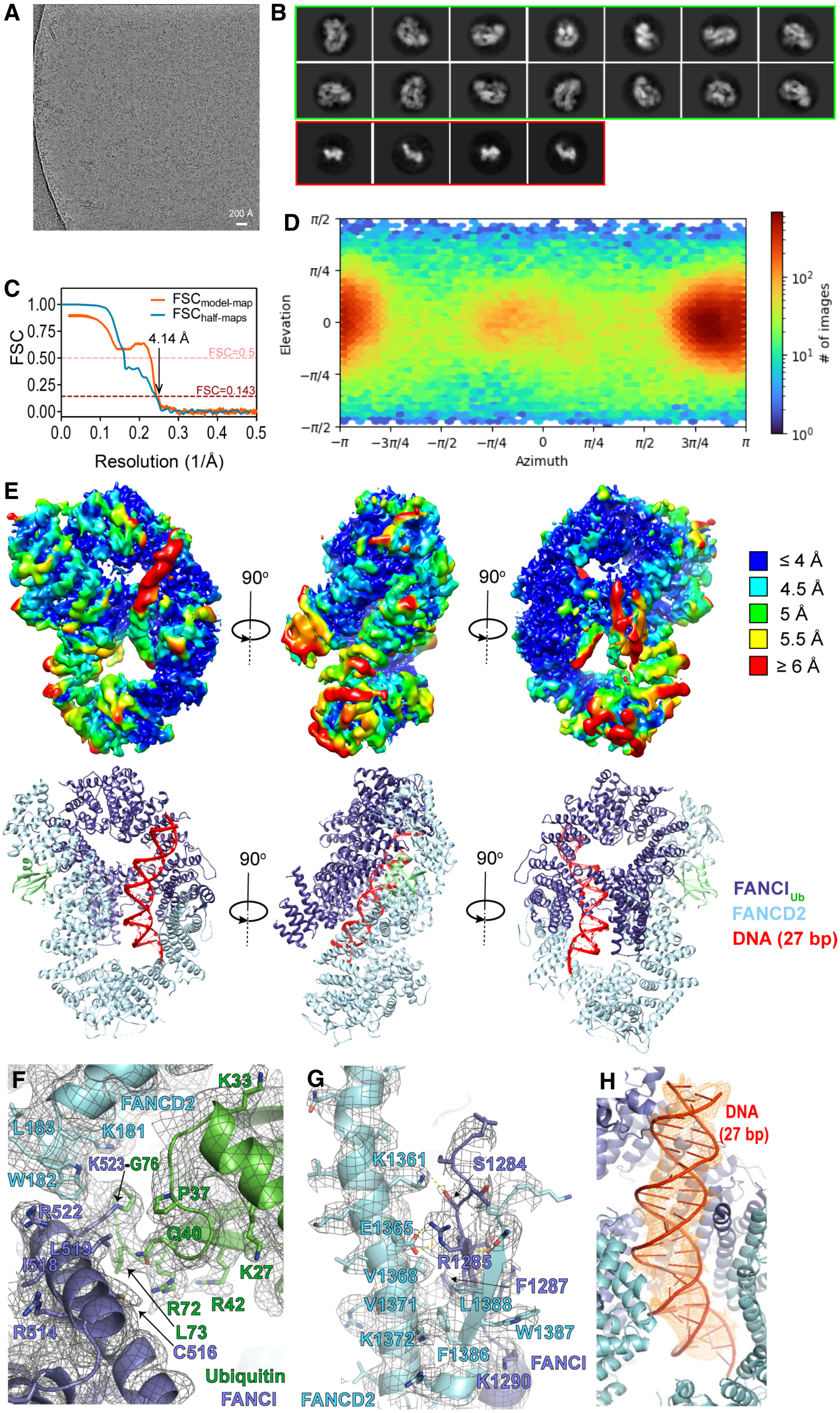
A
200 Å
B
C
FSC
1.00
0.75
0.50
0.25
0.00
0.0 0.1 0.2 0.3 0.4 0.5
Resolution (1/Å)
FSCmodel-map
FSChalf-maps
4.14 Å
FSC=0.5
FSC=0.143
D
Elevation
Azimuth
# of images
E
90°
≤ 4 Å
4.5 Å
5 Å
5.5 Å
≥ 6 Å
FANCIUb
FANCD2
DNA (27 bp)
F
FANCD2
K33
L183
K181
W182
K523-G76
P37
R522
Q40
L519
I518
K27
R72
R42
L73
R514
C516
Ubiquitin
FANCI
G
K1361
S1284
E1365
R1285
V1368
F1287
V1371
L1388
W1387
K1372
F1386
FANCI
K1290
FANCD2
H
DNA
(27 bp)

Figure EV1.

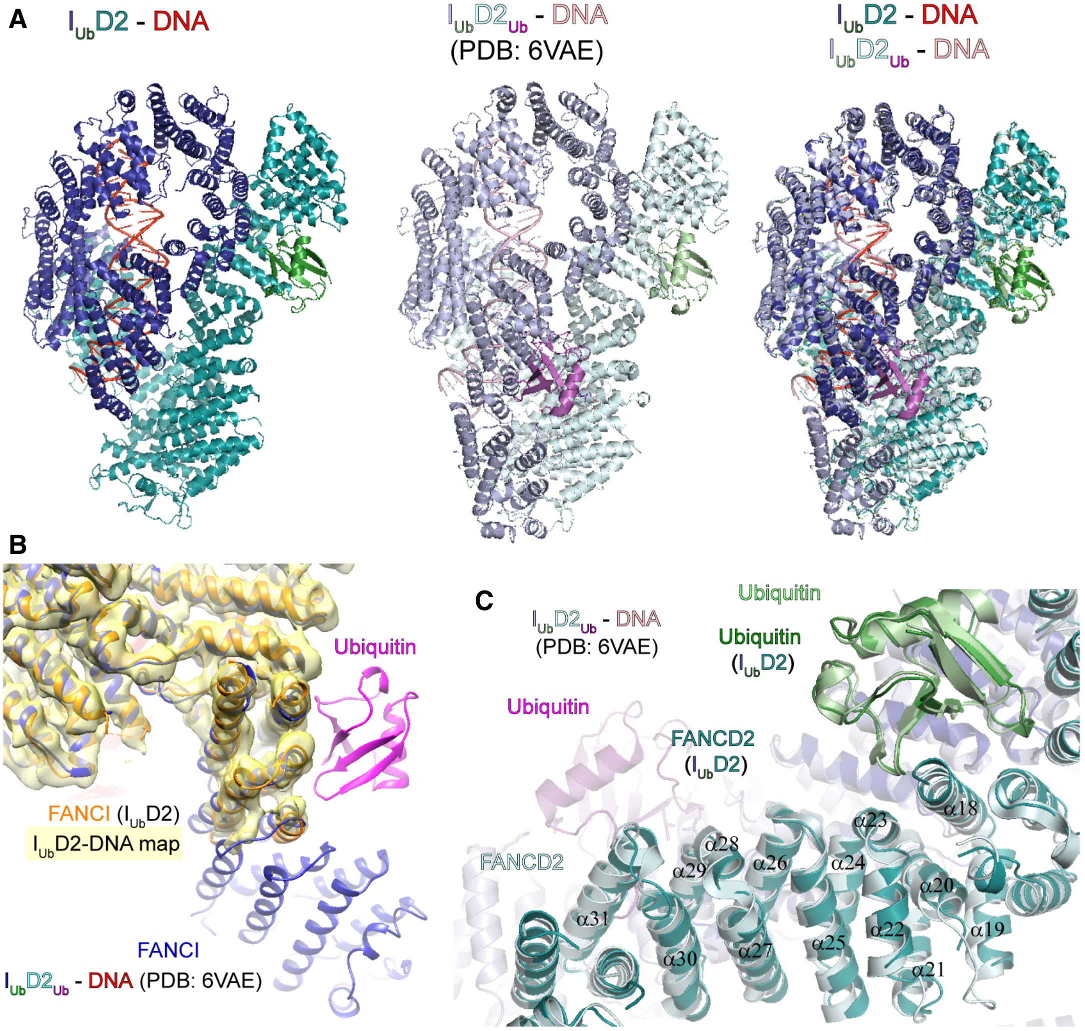

Figure EV2. type="main">I_{Ub}D2-DNA and $I_{Ub}D2_{Ub}$-DNA structure comparison.

A The absence of a FANCD2-conjugated ubiquitin in I_{Ub}D2-DNA structure is associated with a disorder in the N-terminus of FANCI (residues 1–170), when compared with $I_{Ub}D2_{Ub}$-DNA structure. The two structures were aligned in Pymol and visualised from the same angle, either on their own (*left* and *centre*), or together (*right*).

B Helices of FANCI involved in interaction with FANCD2's ubiquitin ($I_{Ub}D2_{Ub}$-DNA structure; FANCI: blue, Ubiquitin: magenta), are positioned differently when the ubiquitin is removed (I_{Ub}D2-DNA structure; FANCI: orange). $I_{Ub}D2_{Ub}$-DNA and I_{Ub}D2-DNA structures were fitted to I_{Ub}D2-DNA sharpened map (yellow) and centred on FANCI N-terminus.

C Removal of ubiquitin (magenta) from FANCD2 results in slight movements affecting several FANCD2 helices, from α31 (helix where ubiquitin is conjugated) up to α18. FANCD2 helices of I_{Ub}D2-DNA and $I_{Ub}D2_{Ub}$-DNA are better aligned towards the N-terminus of FANCD2 (N-terminally to, and including α18 helix of FANCD2), whereby FANCD2 interacts with the ubiquitin (green) conjugated to FANCI. The structures shown in (A), were centred on the central part of FANCD2.

© 2022 The Authors

A

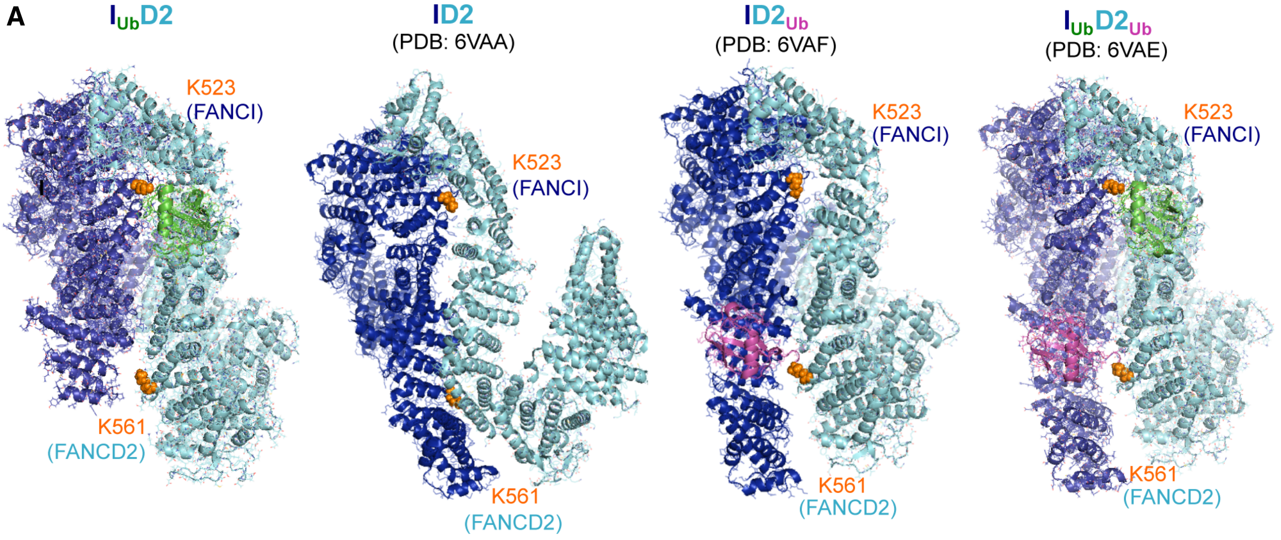

B

	FANCI-K523		FANCD2-K561	
	ID2	$ID2_{Ub}$	ID2	$I_{Ub}D2$
Accessible surface area (Å^2)	153	110	121	117
Burried surface area (Å^2)	48	26	79	0

Figure EV3. FANCI and FANCD2 target lysine positioning and accessibility in DNA-bound $I_{Ub}D2$, ID2, $ID2_{Ub}$ and $I_{Ub}D2_{Ub}$ structures.

A The overall accessibility of FANCI's K523 and FANCD2's K561 is shown within $I_{Ub}D2$-DNA, ID2-DNA (PDB: 6VAA), $ID2_{Ub}$-DNA (PDB: 6VAF) and $I_{Ub}D2_{Ub}$-DNA (PDB: 6VAE) structures. For clarity DNA was removed from the structures. The corresponding lysines are illustrated as orange spheres. $I_{Ub}D2$, $ID2_{Ub}$ and $I_{Ub}D2_{Ub}$ structures were aligned to FANCI of ID2 structure, to allow visualisation of all structures under same orientation.

B FANCI's K523 and FANCD2's K561 accessible surface areas and buried surface area (both in Å^2) are shown in non-ubiquitinated ID2 complex and upon ubiquitination of the other subunit. These values were determined from associated PDB files using the PDBePISA tool (Krissinel & Henrick, 2007) at https://www.ebi.ac.uk/pdbe/pisa/.

© 2022 The Authors

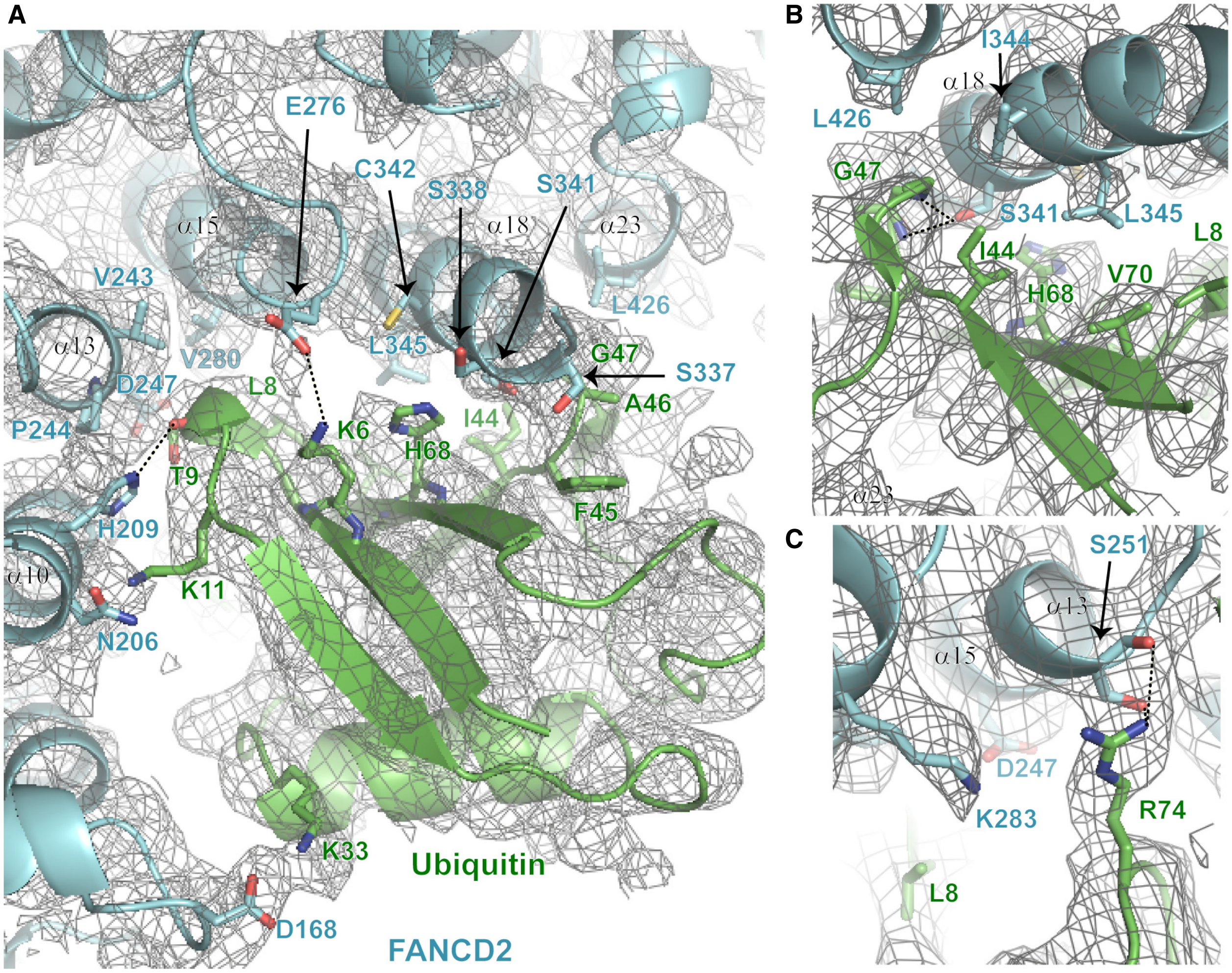

Figure EV4. Cryo-EM density corresponding to the Ubiquitin-FANCD2 interface in the I_{Ub}D2-DNA structure.

A The ubiquitin conjugated to FANCI interacts with several residues located in helices α10, α13, α15, α18 and α23 of FANCD2 in I_{Ub}D2-DNA structure. Assessment of the density map (Phenix auto-sharpened I_{Ub}D2-DNA map) and of the nature and distance between interacting residues, indicate that the ubiquitin of FANCI predominantly interacts with residues located on helices α10 (H209) and α13 (V243 and P244), and on helix α18 (S337, S338, S341, C342) of FANCD2. Interacting residues are indicated and illustrated as sticks. D168 and N206 of FANCD2 (also shown and illustrated as sticks) are positioned in areas corresponding to overlapping density between ubiquitin and FANCD2, but are not sufficiently close to K33 and K11 of ubiquitin, respectively, for a high confidence interaction.

B Ubiquitin-FANCD2 interface from a view centred on residues G47, I44, H68, V70 and L8 of ubiquitin.

C Ubiquitin-FANCD2 interface from a view centred on residues K283 and S251 of FANCD2.

Data information: Dotted straight lines indicate hydrogen bonding.

 © 2022 The Authors
