# Supplementary material for: Structural and biochemical basis of interdependent FANCI‐FANCD2 ubiquitination
Source: EMBO J. 2022 Nov 17;42(3):e111898. doi: 10.15252/embj.2022111898 (PMC9890228; doi:10.15252/embj.2022111898)
Supplement: Supplementary file 4 [file EMBJ-42-e111898-s002.pdf]

THE EMBO JOURNAL

Structural and biochemical basis of interdependent FANCI-FANCD2 ubiquitination

Kimon Lemonidis, Martin Rennie, Connor Arkinson, Viduth Chaugule, Mairi Clarke, James Streetley, and Helen Walden
DOI: 10.15252/embj.2022111898

Corresponding author(s): Kimon Lemonidis (kimon.lemonidis@gmail.com) , Helen Walden (Helen.Walden@glasgow.ac.uk)

Review Timeline:

Transfer from Review Commons:	16th Jun 22
Editorial Decision:	8th Jul 22
Revision Received:	3rd Oct 22
Accepted:	25th Oct 22

Review COMMONS

Editor: Hartmut Vodermaier

Transaction Report:

This manuscript was transferred to The EMBO JOURNAL following peer review at Review Commons.

Review #1

1. Evidence, reproducibility and clarity:

Evidence, reproducibility and clarity (Required)

In this manuscript, Lemonidis et al concentrate on the role of FANCI ubiquitination in the ubiquitination and deubiquitination of the FANCD2 in the FANCI-FANCD2 complex. They work with purified FANCI and FANCD2 proteins that were in vitro ubiquitinated and with purified USP1-UAF1, allowing them to test the kinetics of the ubiquitination and deubiquitination reactions under different conditions. They also show a cryo EM structure of IUbD2 at a resolution of 4.1Å.

Using biochemical assays, the authors show that ubiquitination of one of the subunits (FANCI or FANCD2) enhances the ubiquitination of the other. D2Ub can be de-ubiquitinated with high efficiency, with no protection by DNA and FANCI. However, when FANCI is ubiquitinated, FANCD2 de-ubiquitination is decreased. It is also more easily re-ubiquinated. The efficiency of de-ubiquitination of FANCIUb is strongly decreases when complexed to DNA and FANCD2, and it is independent of the FANCD2 ubiquitination. The structure shows that the IUbD2, like IUbD2Ub and ID2 Ub, is clamped on the DNA, but the FANCD2K561 site in exposed explaining how re-ubiquitination of the FANCD2 is facilitated.

Major comments:

The key conclusions are overall convincing. The IUbD2 structure shows a conformation in which the FANCD2 lysine is more easily accessible than in ID2 (3B). Biochemical assays support the claim that the ubiquitination of FANCI and FANCD2 promote the ubiquitination of their opposing subunit in ID2 (FANCD2, and FANCI, respectively). Furthermore, robust biochemical assays and structures in Figure 4 support the claim that the ubiquitin on FANCI is relatively more protected than the ubiquitin on FANCD2, of which the latter can be de-ubiquitinated with high efficiency.

1. The major caveat is that these structures were generated using "engineered Ube2T and SpyCatcher-SpyTag", and not via the FA core complex. Recent structural work by the Passmore and Pavletich labs shows the intricate process of ID2 complex ubiquitination, and propose a sequential ubiquitination model that is tied closely to the unique structural properties of the FA core and the ID2 interface. Similarly, the

biochemistry is done in the absence of the core complex which may change the kinetics of the ubiquitination and deubiquitination. So, although the biochemical experiments performed are robust, these caveats need to be explicitly noted in the manuscript.
2. Ideally, the authors should also solve cryo EM structure of IUbD2Ub and compare this to 6VAE to show that their in vitro ID2 assembly leads to structures aligning with the FA-core complex ubiquitinated form of ID2.
3. I would suggest including ubiquitinated FANCD2 in the PIFE experiments in Figure 2, to assess differential binding affinity of I and IUb. This would help serve as a control that FANCD2 ubiquitination also strengthens the ID2 complex binding the DNA.
4. The authors claim that "ubiquitination of either of the two subunits of ID2 (FANCI or FANCD2) actually favors ubiquitination of the other subunit" (supported by biochemical and structural data in Figure 3). In Figure 3A-B, it is difficult to draw a conclusion on the "accessibility of the lysine residues" from these figures. Authors should include a measurement of the distances between the Lysines and the NTD of the opposite subunit. This would add a numeric measure to "accessibility".
5. Wang et al, 2020 show that the FANCI K523 is only accessible for ubiquitination upon prior FANCD2 ubiquitination. However, the IUbD2 state could be formed by FANCD2-deubiquitination of IUbD2Ub. Figure 5 may need to changed to remove the arrow ID2 state to IUbD2.
6. In Figure 3B, the structure of IUbD2Ub should be included to allow a proper comparison of the Lysine accessibility of IUbD2 and ID2Ub versus IUbD2Ub.

Minor comments:

1. Please include specific method of expressing, purifying and ubiquitinating FANCI and FANCD2. This method doesn't become clear by referencing previous papers, and these methods might have changed.
7. The final paragraph needs to be reworded. The following sentence is very confusing "Of these two interfaces, one is absent in equivalent ubiquitin-FANCI interactions in ID2Ub-DNA structure (Wang et al, 2020)(Fig. 4D-E)." It does not come across that ubiquitin of FANCIUb lacks an interface with FANCD2.
2. "Our structure indicates that this ubiquitin-FANCD2 interface ..." probably should be "Our structure indicates that the interface between FANCD2 and the ubiquitin of FANCIUb ..."
3. I would suggest adding additional supplemental figures of Figure 3B including 1 extra angle for each structure. This would help gauge "Lysine accessibility".
4. Please add PDB images in Figure 4D from the IUbD2Ub structure of both the interface between FANCD2 and ubiquitin of FANCIUb, and FANCI and the ubiquitin of FANCD2Ub. This is important to allow for the comparison of the different

ubiquitin interfaces amongst the IUbD2Ub, ID2Ub, and IUbD2 structures

References:

Previous in vitro and in vivo data well referenced in introduction and discussion.

Statistics:

Satisfactory

Comments on time and resources:

Solving the cryo EM structure of IUbD2Ub is not a trivial ask, but the authors may already have it or may be inclined to do it since they already have access to purified IUb D2Ub and have optimized cryo EM conditions for IUbD2 in this paper. The remaining suggestions seems to be minor (repeat of biochemical experiment, measurement of pre-existing data, and minor changes to figures).

2. Significance:

Significance (Required)

This work fits very well with the recent structural advances describing ubiquitination and de-ubiquitination of the Fanconi ID2 complex: Wang et al. 2020 and 2021, Alcon et al. 2020. These papers were groundbreaking in describing the mechanism in which FANCD2 and FANCI are ubiquitinated by the FA core complex. The same group (Rennie et al. 2021) solved a structure of ID2 bound to USP1-UAF1 which was also important for understanding of the Fanconi anemia pathway function. The current manuscript sheds light on the role of the Ubiquitinated FANCI in the function of the FANCID2 dimer.

The manuscript will be of interest to the DNA repair, genome integrity, hereditary diseases and ubiquitin fields. This review was written by an investigator in the genome integrity and Fanconi anemia field.

3. How much time do you estimate the authors will need to complete the suggested revisions:

Estimated time to Complete Revisions (Required)

(Decision Recommendation)

Between 1 and 3 months

4. Review Commons values the work of reviewers and encourages them to get credit for their work. Select 'Yes' below to register your reviewing activity at Publons; note that the content of your review will not be visible on Publons.

Reviewer Publons

Yes

Review #2

1. Evidence, reproducibility and clarity:

Evidence, reproducibility and clarity (Required)

The manuscript by Lemonidis et al describes a structural and biochemical basis for the interdependency of FANCI-FANCD2 monoubiquitylation. Continuing their landmark work in the FANCI-FANCD2-Ub Cryo-EM structure, this study shows that the homeostasis of FANCI-Ub-FANCD2-Ub (ID2-diUb) can be partly explained by accessibility differences of the ubiquitylated forms of FANCI and FANCD2 complex. Using structure and enzymatic assays, the authors show that FANCI-Ub is not as susceptible for deubiquitylation by USP1-UAF1 deubiquitinase (DUB) when in complex with FANCD2 and DNA. In contrast, FANCD2 deubiquitiylation is more efficient when FANCI is unmodified, and more protected when it is in complex with FANCI-Ub and DNA. Interestingly, FANCI ubiquitylation progresses at a faster rate when in complex with FANCD2-Ub and DNA. Even though this is somewhat an incremental study, nevertheless, the mechanisms of USP1 can or cannot remove ubiquitylated FANCI-FANCD2-DNA complex is intriguing and opens new areas of study to understand whether several different states of modified and unmodified FANCI-FANCD2 exists physiologically or not and what functional differences they may have.

The major limitions of this study is the lack of functional connection to the described mechanisms of deubiquitylation or reubiquitylation of FANCI-FANCD2 complexes. In the future, hopefully, this group or others will generate point mutants that can selectively separate the functions of FANCI or FANCD2 deubiquitylation or FANCI or FANCD2 reubiquitylation to show the importance of dynamic regulation of these processes in a cellular or in vivo context.

A major comment is whether the authors can by structural inference figure out what part of USP1 N-terminal extension is required for interaction and recognition of FANCI-FANCD2-Ub-DNA complexes. This is the only part of the study that wasn't very satisfying. Perhaps the authors can address this with some modeling and/or with additional mutational analyses on USP1.

One minor comment is that the authors only cite the Smorgorzewska et al, Cell, 2007 paper for observing the first interdependency of FANCI-FANCD2 ubiquitylation in cells (Citations in the Intro and Discussion sections). The authors should also cite Sims et al NSMB, 2007 for the exact same observation shown in the two papers just to be consistent.

2. Significance:

Significance (Required)

Significance of the study is as indicated above.

The audience is the Fanconi Anemia and ubiquitin field.

3. How much time do you estimate the authors will need to complete the suggested revisions:

Estimated time to Complete Revisions (Required)

(Decision Recommendation)

Less than 1 month

4. Review Commons values the work of reviewers and encourages them to get credit for their work. Select 'Yes' below to register your reviewing activity at Publons; note

that the content of your review will not be visible on Publons.

Reviewer Publons

Yes

Review #3

1. Evidence, reproducibility and clarity:

Evidence, reproducibility and clarity (Required)

Summary:

Lemonidis et al perform a structural and biochemical investigation of FANCI-Ub/FANCD2 complex. The key findings are that FANCD2-Ub is more rapidly deubiquitinated that FANCI-Ub by USP1:UAF1, particularly in the context of the full FANCD2-Ub:FANCI-Ub complex. They present a structure of FANCI-Ub/FANCD2 and show key features that distinguish it from the other FA core complex structures. Several regions are unstructured in their model, which the authors propose is because of the absence of ubiquitin on FANCD2. The exposure of Ubiquitinated lysine of FANCD2 is suggested to provide a mechanism for the rapid reubiquitination of this site when bound to FANCI-Ub, and they also show this biochemically as a faster ubiquitination of FANCD2 when bound to FANCI-Ub instead of FANCI-Ub. A very neat conclusion is made about the dual role of FANCI-Ub in maintaining FANCD2-Ub in a DNA clamped state.

Major comments:

No major issues identified. The data supports the conclusions. Some mutagenesis studies that investigate the hypotheses generated by the structure would increase the overall impact of the study.

Minor comments:

The role of FANCI-Ub and DNA binding in protecting both FANCI and FANCD2

from deubiquitination was first shown in van Twest et al 2017 Molecular Cell, however this is not referred to in the manuscript.

USP1:UAF1 has been shown to have DNA binding activity (eg Liang et al 2016 Cell Reports). Could this DNA binding activity potentially reduce its activity on I-Ub-D2-Ub in the presence of DNA (ie through sequestration away from the substrate by binding to other DNA?). This could be tested by showing that titrating the amount of DNA in the reaction does not inhibit deubiquitination. Also, one previous study showed that DNA *stimulates* deubiquitination of FANCD2. This study used complexes where FANCI was not ubiquitinated, but is a different result to what is seen in Figure 4A (but perhaps because deubiquitination is already 100% in the absence of DNA in these experiments). Some investigation/discussion of this discrepancy would be good to include.
Some further discussions speculating why a FANCI-Ub is necessary in the FA pathway (ie why not just have FANCD2-Ub as the clamping mechanism given that it is sufficient to lock the protein onto DNA?) would increase the interpretation of the work by non-specialists.

2. Significance:

Significance (Required)

The work provides a significant advance in our understanding of the mechanism by which di-monoubiquitinated FANCD2:FANCI clamps the complex onto DNA. The first structures of I-UbD2 are presented, and some evidence for why they exist in cells (as an intermediate state of deubiquitination of the complex). Previous studies already showed that FANCI-Ub and DNA binding prevent the deubiquitination activity (eg van Twest et al 2017, Arkinson et al 2018, Rennie et al 2020, Wang et al 2020) of USP1:UAF1.

The work has clinical relevance - USP1:UAF1 inhibitors and FA core complex inhibitors are being developed by several biotech for cancer therapy (this is not referenced in the manuscript, and could perhaps be included in the introduction?).
Researchers in several fields will be interested in the work: those working on FA pathway and mechanistic understanding of FANCD2 monoubiquitination, as well as those working more generally in ubiquitination and deubiquitination pathways. The structures included "complete the set" for all states of FANCD2:FANCI ubiquitination states, and this is a highly cited field.
My expertise is in understanding the mechanistic basis of FANCD2 and FANCI ubiquitination and deubiquitination so I am well suited to evaluate all aspects of the proposal.

3. How much time do you estimate the authors will need to complete the suggested revisions:

Estimated time to Complete Revisions (Required)

(Decision Recommendation)

Less than 1 month

4. Review Commons values the work of reviewers and encourages them to get credit for their work. Select 'Yes' below to register your reviewing activity at Publons; note that the content of your review will not be visible on Publons.

Reviewer Publons

Yes

Revision Plan

Manuscript number: RC-2022-01411
Corresponding author(s): Kimon, Lemonidis

[The "revision plan" should delineate the revisions that authors intend to carry out in response to the points raised by the referees. It also provides the authors with the opportunity to explain their view of the paper and of the referee reports.

The document is important for the editors of affiliate journals when they make a first decision on the transferred manuscript. It will also be useful to readers of the reprint and help them to obtain a balanced view of the paper.

If you wish to submit a full revision, please use our "Full Revision" template. ***It is important to use the appropriate template to clearly inform the editors of your intentions.****]*

1. General Statements [optional]

This section is optional. Insert here any general statements you wish to make about the goal of the study or about the reviews.

We would like to thank the reviewers for their useful comments and suggestions. We have now applied changes to the text and figures based on these, while we also plan to do few more experiments to address some of the points raised. We hope that these changes will substantially improve the current manuscript and we are grateful to the 3 reviewers for their contribution.

Regarding the scope of this study, our aim was to explain, by structural and biochemical means, the interdependency in ID2 ubiquitination observed before. We do hope to perform some functional studies in the future, but this would be the aim of another study, and should ideally occur once the mechanism of FANCI ubiquitination/deubiquitination is better elucidated.

Regarding the analyses for which we are unable to perform for revision, or which might be unnecessary, these refer to 3 points raised. We have previously produced structures of the di-mono-ubiquitinated and FANCD2-ubiquitinated ID2 complex, using our complex reconstitution approach (Rennie et al 2020, EMBO Reports; Rennie et al, 2021; Nature Structural and Molecular Biology). All these structures had the same closed-on-DNA conformation as the structures produced by other groups using the FA-core complex. Hence, we feel that repeating our previous structural work would be unnecessary. Unfortunately, we are currently not set-up for co-expression of all FA-core components for purification of the entire FA-core complex (similarly to what performed in Pavletich's and Passmore's group). However, we also rationalize in our response that, both our structures, as well as the biochemistry of ID2 ubiquitination and deubiquitination, are consistent with what has been observed before with FA-core complex and

the FA-core mechanism of action. Lastly, we explain that preferential binding of USP1-UAF1 to DNA would be inconsistent with ternary complex (USP1-UAF1-ID2$_{Ub}$-DNA) formation (Rennie et al 2021, Nature Structural and Molecular Biology). Therefore, DNA is unlikely to sequester USP1-UAF1 away from ID2. DNA is much more likely to inhibit ID2 deubiquitination by stabilizing the closed ID2 conformation, as evidence suggest.

2. Description of the planned revisions

Insert here a point-by-point reply that explains what revisions, additional experimentations and analyses are planned to address the points raised by the referees.

Reviewer #1

3. I would suggest including ubiquitinated FANCD2 in the PIFE experiments in Figure 2, to assess differential binding affinity of I and IUb. This would help serve as a control that FANCD2 ubiquitination also strengthens the ID2 complex binding the DNA.

We intend to carry out the proposed PIFE experiments. Although we have previously shown that FANCD2 ubiquitination enhances ID2 binding to dsDNA (Rennie et al 2020, EMBO Reports), we appreciate that affinities determined in that work, were not measured with excess D2/D2$_{Ub}$. Hence, we agree with the reviewer that PIFE assays with excess D2$_{Ub}$ (instead of D2) would be a good control to include for direct comparison with affinities measured in Figure 2.

Reviewer #3

Some mutagenesis studies that investigate the hypotheses generated by the structure would increase the overall impact of the study.

We agree. We intend to mutate key FANCD2 residues involved in binding with the ubiquitin conjugated to FANCI, to reduce this interaction. Our aim is to produce a comparable interaction with the one observed between FANCI and the ubiquitin conjugated to FANCD2. Deubiquitination assays will be subsequently carried out to assess whether the presence of this mutant FANCD2 still protects from FANCI deubiquitination. This would inform us on whether or not the respective ubiquitin-FANCD2 interface is the (sole) reason for the increased resistance of FANCI-ubiquitinated ID2 complexes to deubiquitination.

3. Description of the revisions that have already been incorporated in the transferred manuscript

Please insert a point-by-point reply describing the revisions that were already carried out and included in the transferred manuscript. If no revisions have been carried out yet, please leave this section empty.

Revision Plan

Reviewer #1

1. The major caveat is that these structures were generated using "engineered Ube2T and SpyCatcher-SpyTag", and not via the FA core complex. Recent structural work by the Passmore and Pavletich labs shows the intricate process of ID2 complex ubiquitination, and propose a sequential ubiquitination model that is tied closely to the unique structural properties of the FA core and the ID2 interface. Similarly, the biochemistry is done in the absence of the core complex which may change the kinetics of the ubiquitination and deubiquitination. So, although the biochemical experiments performed are robust, these caveats need to be explicitly noted in the manuscript.

We agree that the kinetics of ubiquitination may be somewhat different when an engineered UBE2T is used along with a truncated FANCL, as opposed to wild type UBE2T and FA-core complex. However, we have previously shown that our approach results in site specific modification of FANCD2 and FANCI (Chaugule et al 2019 Methods in Enzymology; Chaugule et al 2020 Nature Chemical Biology) and in ID2 complexes having the same closed-on-DNA conformation (Rennie et al 2020 EMBO reports; Rennie et al 2021; Nature Structural and Chemical Biology) as the one observed with complexes produced via wild-type UBE2T and FA-core complex (Alcon et al 2020 Nature Structural and Chemical Biology; Wang et al 2020 Nature). This suggests that ID2 ubiquitination is actually responsible for both producing and maintaining the final ID2 conformation. Hence the conformation of our complex would also be determined by its ubiquitinated status. Accordingly, our deubiqutination assays would be unaffected by the way our ubiquitinated complexes were formed, since these are structurally indistinguishable from complexes produced using FA-core catalyzed reactions. Regarding the biochemistry experiments, we should note that wild-type tag-less ubiquitin was used in our *in vitro* ubiquitination reactions, whereas the tag on ubiquitin (GST-spyCatcher-Spytag or GST-tag) had been removed during preparation of ubiquitinated FANCI/FANCD2, and hence our deubiqutination assays also occurred on tag-less ubiquitin. Lastly, irrespective of any differences in kinetics between the two ubiquitination approaches, our results are in agreement with what had been observed also with FA-core: FANCI ubiquitination progresses at a much faster rate once FANCD2 is ubiquitinated. Although we have no FA-core catalyzed control for I_{Ub}D2 vs ID2 ubiquitination, we do not see any reason why FANCD2 would not be ubiquitinated faster in I_{Ub}D2 complex in that case: FA-core has been shown to ubiquitinate FANCD2 by altering the conformation of the ID2 complex to a closed-on-DNA state to achieve catalysis. In I_{Ub}D2 complex this step can be bypassed, since this complex is already in the closed conformation, and hence the reaction is expected to progress faster.

To reflect the above, we have modified the part where we describe how we created the I_{Ub}D2-DNA complex (in Results section) into: "*To address these questions, we used our in vitro reconstitution approach (Arkinson et al, 2018; Rennie et al, 2020, 2021) to assemble a 1:1:1 I_{Ub}D2-DNA complex, from purified I_{Ub}, D2 and dsDNA (61 bp long), and subsequently determined its structure by cryo-EM. Such reconstitution approach has been successfully applied to produce ID2$_{Ub}$/I_{Ub}D2$_{Ub}$-DNA structures (Rennie et al, 2020) and USP1-UAF1-bound ID2$_{Ub}$-DNA structures (Rennie et al, 2021). These complexes have been shown to adopt the same closed ID2 conformation as the one observed in ubiquitinated ID2-DNA complexes produced following FA-core-catalyzed ID2 ubiquitination (Alcón et al, 2020; Wang et al, 2020).*

This suggests that, despite the requirement of FA-core for opening up the ID2 complex for subsequent ubiquitination (Wang et al, 2021), ID2 ubiquitination is actually required for both producing and maintaining the final closed ID2 conformation. Hence, we reasoned that our in vitro assembled complex would also be structurally indistinguishable from a complex produced through removal of FANCD2's ubiquitin from $I_{Ub}D2_{Ub}$."

We have also included an additional paragraph in discussion in support of our findings shown in Fig. 3C-D, quoting agreements with previous *in vitro* assays using FA-core, as well as with the mechanism of FA-core action (Wang et al 2021, Nature Structural and Molecular Biology). In this paragraph, we also give an analogy whereby USP1-UAF1 catalyzed ID2 deubiquitination is also accelerated, when the closed ID2 state is compromised (Wang et al 2020, Nature)

4. The authors claim that "ubiquitination of either of the two subunits of ID2 (FANCI or FANCD2) actually favors ubiquitination of the other subunit" (supported by biochemical and structural data in Figure 3). In Figure 3A-B, it is difficult to draw a conclusion on the "accessibility of the lysine residues" from these figures. Authors should include a measurement of the distances between the Lysines and the NTD of the opposite subunit. This would add a numeric measure to "accessibility".

We have now modified panel B of Figure 3 to better illustrate how FANCI/FANCD2 lysine accessibility improves when the other subunit is ubiquitinated. We specifically show for each of the two lysines (epsilon amino group), all the residues (side-chains) of the other subunit located within 8 Å, and how the distance to the closest residue increases upon ubiquitination of the other subunit. We have additionally incorporated a table (Fig. EV3B) displaying accessible/buried surface areas for these lysines for all available ID2 complexes.

5. Wang et al, 2020 show that the FANCI K523 is only accessible for ubiquitination upon prior FANCD2 ubiquitination. However, the IUbD2 state could be formed by FANCD2-deubiquitination of IUbD2Ub. Figure 5 may need to changed to remove the arrow ID2 state to IUbD2.

We have now removed this arrow in that Figure (which is now Figure 6 in the manuscript).

6. In Figure 3B, the structure of IUbD2Ub should be included to allow a proper comparison of the Lysine accessibility of IUbD2 and ID2Ub versus IUbD2Ub.

We have now incorporated in Fig 3B for comparison, images of $I_{Ub}D2_{Ub}$ aligned with the FANCD2 of $I_{Ub}D2$ (left panel), or with FANCI of $ID2_{Ub}$ (right panel).

7. The final paragraph needs to be reworded. The following sentence is very confusing "Of these two interfaces, one is absent in equivalent ubiquitin-FANCI interactions in ID2Ub-DNA structure (Wang et al, 2020)(Fig. 4D-E)." It does not come across that ubiquitin of FANCIUb lacks an interface with FANCD2.

We agree that there may be some confusion relating to the identity of ubiquitin and corresponding complex when these interfaces are mentioned and compared. We have thus modified this paragraph to: "Whereas the FANCI interaction with the ubiquitin conjugated to FANCD2 has no protective role against USP1-UAF1 mediated FANCD2 deubiquitination, the interaction of FANCI's ubiquitin with FANCD2 (in $I_{Ub}D2$-DNA and $I_{Ub}D2_{Ub}$-DNA complexes)

efficiently protects against FANCI deubiquitination. In each case, ubiquitin's hydrophobic I44 patch is involved in interaction with the other ID2 subunit; however, the ubiquitin conjugated to FANCI forms a more extended interface with FANCD2 (Wang *et al*, 2020)(Fig. 5A). This extended interface is formed predominantly via hydrophobic interactions of residues H209 (α10 helix), V243, P244 and D247(α13 helix) of FANCD2 with residues T9 and K11 of ubiquitin, and is further stabilized by hydrogen bonding between ubiquitin's T9 and R74 with FANCD2's H209 and S251 (or E217) , respectively (Fig. 5A). The residues of FANCD2 predicted to be involved in this extended interface...*"*

Minor

1. Please include specific method of expressing, purifying and ubiquitinating FANCI and FANCD2. This method doesn't become clear by referencing previous papers, and these methods might have changed.

We have now given a brief description of the methods employed for producing ubiquitinated and non-ubiquitinated versions of FANCI/FANCD2 and related ID2 complexes, and also of methods employed for producing USP1, USP1ΔN, UAF1 and related complexes.

2. "Our structure indicates that this ubiquitin-FANCD2 interface ..." probably should be "Our structure indicates that the interface between FANCD2 and the ubiquitin of FANCIUb ..."

We have reworded the whole paragraph as mentioned above. This is now changed into: *"This extended interface is formed predominantly via hydrophobic interactions of residues* …"

3. I would suggest adding additional supplemental figures of Figure 3B including 1 extra angle for each structure. This would help gauge "Lysine accessibility".

Figure 3B now consists of focused images of FANCD2-K561 and FANCI-K523 region, showing their accessibility in ID2, $I_{Ub}D2$, $ID2_{Ub}$ and $I_{Ub}D2_{Ub}$ structures (with residues of the other subunit within 8Å and measurements of lysine-NTD distances shown). The comparison of entire ID2, $I_{Ub}D2$, $ID2_{Ub}$ and $I_{Ub}D2_{Ub}$ structures shown before, is now moved to Fig. EV3A, with extra numerical details of lysine accessibility shown in Fig. EV3B.

4. Please add PDB images in Figure 4D from the IUbD2Ub structure of both the interface between FANCD2 and ubiquitin of FANCIUb, and FANCI and the ubiquitin of FANCD2Ub. This is important to allow for the comparison of the different ubiquitin interfaces amongst the IUbD2Ub, ID2Ub, and IUbD2 structures

We have now included illustrations of respective ubiquitin-FANCI and ubiquitin-FANCD2 interactions in $I_{Ub}D2_{Ub}$ structure for direct comparison. Due to the increased length of information shown (with results from extra experiments to be also included in the final revision), these, along with the amino-acid conservation subfigure, are now illustrated in a separate figure (Figure 5). Upon closer inspection of FANCI interactions with the ubiquitin on FANCD2 in $I_{Ub}D2_{Ub}$-DNA complex, we realized that E34 of ubiquitin had been wrongly assigned to interact with K83 of FANCI in Fig. EV2. These residues are estimated to be 4.3 Å apart, and had not been reported to interact with each other in either this structure or in $ID2_{Ub}$-DNA structure (Wang *et al* 2020, Nature). Hence, we have now corrected for this mistake in Fig. EV2 and have removed the

sentence “*A crucial role for the stabilization of the FANCI’s N-terminus in the $I_{Ub}D2_{Ub}$-DNA complex might be FANCI’s K83 interaction with E34 of ubiquitin (Fig. EV2B)*” from the text, since such weak binding is unlikely to stabilize FANCI’s N-terminus. We have also added the word “predominantly” for residues involved in interactions in Fig. EV2, since there are few more residues (shown in Fig. 5A) that participate in the FANCI-ubiquitin interaction, not shown in Fig. EV2.

Reviewer #2

The major limitions of this study is the lack of functional connection to the described mechanisms of deubiquitylation or reubiquitylation of FANCI-FANCD2 complexes. In the future, hopefully, this group or others will generate point mutants that can selectively separate the functions of FANCI or FANCD2 deubiquitylation or FANCI or FANCD2 reubiquitylation to show the importance of dynamic regulation of these processes in a cellular or in vivo context

The scope of this work was in providing a biochemical and structural explanation of the interdependency in FANCI-FANCD2 ubiquitination previously observed *in vivo*. Providing a functional connection of some of our *in vitro* and structural findings, however, is a long term aim in our group. Nevertheless, we believe that important aspects relating to the mechanism of FANCI ubiquitination and deubiquitination need to be uncovered first to allow generation of mutants that selectively affect ubiquitination and deubiquitination of each of the two ID2 subunits. To address the importance of unravelling the mechanism of FANCI ubiquitination and deubiquitination for future *in vivo* studies, we have a created a separate paragraph highlighting this in the discussion.

A major comment is whether the authors can by structural inference figure out what part of USP1 N-terminal extension is required for interaction and recognition of FANCI-FANCD2-Ub-DNA complexes. This is the only part of the study that wasn't very satisfying. Perhaps the authors can address this with some modeling and/or with additional mutational analyses on USP1.

We have previously shown that residues R22 and L23 of USP1 N-terminus are involved in FANCD2 binding (Arkinson, et al 2018, Life Science Alliance), which is supported by recently AlphaFold simulations performed in our group (Rennie et al 2022; bioRxiv). These are now stated in the Results section, just after the sentence: *“The above suggest that the interaction of FANCD2’s ubiquitin with FANCI (in the $I_{Ub}D2_{Ub}$-DNA complex) does not protect against FANCD2 deubiquitination, as the latter can be efficiently achieved by a mechanism that involves USP1’s N-terminus”.*

One minor comment is that the authors only cite the Smorgorzewska et al, Cell, 2007 paper for observing the first interdependency of FANCI-FANCD2 ubiquitylation in cells (Citations in the Intro and Discussion sections). The authors should also cite Sims et al NSMB, 2007 for the exact same observation shown in the two papers just to be consistent.

The reason that this article was not referenced in instances where this interdependency was mentioned, was because that study did not show that FANCD2 ubiquitination is dependent on

FANCI ubiquitination, but rather that loss of FANCI (or certain FANCI mutations) disrupt FANCD2 ubiquitination. Regardless, we agree that this study should be given credit and be cited along with the Smorgorzewska et al, Cell, 2007 paper, for showing that FANCI ubiquitination depends on FANCD2 ubiquitination and for providing evidence which are consistent with the interdependency observed by Smorgorzewska et al. We have now included this reference in parts where interdependent FANCI-FANCD2 ubiquitination is mentioned.

Reviewer #3

Minor comments: The role of FANCI-Ub and DNA binding in protecting both FANCI and FANCD2 from deubiquitination was first shown in van Twest et al 2017 Molecular Cell, however this is not referred to in the manuscript.

To incorporate this, we have now altered the part where the protective role of DNA is mentioned in the results section, into: *"Previous work has shown that DNA is required for efficient protection of both FANCI and FANCD2 against USP1-UAF1 mediated deubiquitination (van Twest et al, 2017; Arkinson et al, 2018). Focusing on FANCD2 deubiquitination, we have previously found that FANCI ubiquitination (but not FANCI itself) is additionally required for restricting FANCD2 deubiquitination, but has no effect in protecting from FANCD2 deubiquitination, in the absence of dsDNA (Arkinson et al, 2018)."*

Also, one previous study showed that DNA *stimulates* deubiquitination of FANCD2. This study used complexes where FANCI was not ubiquitinated, but is a different result to what is seen in Figure 4A (but perhaps because deubiquitination is already 100% in the absence of DNA in these experiments). Some investigation/discussion of this discrepancy would be good to include. USP1:UAF1 has been shown to have DNA binding activity (eg Liang et al 2016 Cell Reports). Could this DNA binding activity potentially reduce its activity on I-Ub-D2-Ub in the presence of DNA (ie through sequestration away from the substrate by binding to other DNA?).

Although we cannot exclude the possibility that such discrepancies may derive from differences in the way the respective protein/complexes were produced, the main difference between our assays and theirs, is in the type of DNA used (ssDNA vs dsDNA). It is thus likely that ubiquitinated ID2 complexes produced by a DNA structure other than dsDNA may be more amenable to deubiquitination by USP1-UAF1. Otherwise our results with dsDNA are very reproducible and also in agreement with what has been reported by another group (van Twest et al, 2017). We have thus changed DNA into dsDNA in several parts of the discussion to distinguish it from ssDNA (or other DNA structures) and modified the discussion a bit to also include the following sentences regarding the work of Liang et al 2016, 2019 (4th paragraph): *"Interestingly, the opposite effect (DNA promoting USP1-UAF1-mediated FANCD2 deubiquitination) has been reported in a study utilizing a ~60% FANCD2-ubiquitinated ID2 complex produced with the aid of a 64-mer single-stranded DNA (Liang et al, 2019). While the reasons for such discrepancy need to be further investigated, it is likely that the source of DNA (singe- versus double- stranded) used for ID2 ubiquitination, determines whether FANCD2 deubiquitination will be promoted or inhibited. UAF1 has been shown to bind both double-*

stranded (dsDNA), single-stranded (ssDNA), or more complex D-loop structures of DNA, in vitro (Liang et al, 2016, 2019). Although we cannot exclude the possibility of a UAF1-ssDNA binding event promoting ID2 deubiquitination, there is no evidence in support of dsDNA-UAF1 binding influencing ID2 deubiquitination: cryo-EM analysis of in vitro assembled USP1-UAF1-ID2$_{Ub}$-DNA complexes, suggests that formation of a ternary complex is favored, with both dsDNA and USP1-UAF1 preferentially binding ubiquitinated ID2, rather than each other (Rennie et al, 2021)."

Some further discussions speculating why a FANCI-Ub is necessary in the FA pathway (ie why not just have FANCD2-Ub as the clamping mechanism given that it is sufficient to lock the protein onto DNA?) would increase the interpretation of the work by non-specialists.

Our work shows that FANCI ubiquitination is required for maintaining the ID2 clamping on DNA, which would otherwise be rapidly lost through deubiquitination of FANCD2. To elaborate this we have added to the Discussion (end of second paragraph) the following sentences: *"…, and therefore ubiquitinated ID2 cannot easily revert to a non-ubiquitinated state. In essence, FANCI ubiquitination, via maintaining FANCD2 ubiquitination, commits the ID2$_{Ub}$ complex for FA repair, since without FANCI ubiquitination such complex would be rapidly transformed to a non-ubiquitinated ID2 complex, through the action of USP1-UAF1."*

The work has clinical relevance - USP1:UAF1 inhibitors and FA core complex inhibitors are being developed by several biotech for cancer therapy (this is not referenced in the manuscript, and could perhaps be included in the introduction?).

We have now included text and references highlighting the clinical relevance of this study in the penultimate paragraph of our introduction. We thank the reviewer for point out this.

4. Description of analyses that authors prefer not to carry out

Please include a point-by-point response explaining why some of the requested data or additional analyses might not be necessary or cannot be provided within the scope of a revision. This can be due to time or resource limitations or in case of disagreement about the necessity of such additional data given the scope of the study. Please leave empty if not applicable.

Reviewer #1

2. Ideally, the authors should also solve cryo EM structure of IUbD2Ub and compare this to 6VAE to show that their in vitro ID2 assembly leads to structures aligning with the FA-core complex ubiquitinated form of ID2.
Solving the cryo EM structure of IUbD2Ub is not a trivial ask, but the authors may already have it or may be inclined to do it since they already have access to purified IUb D2Ub and have optimized cryo EM conditions for IUbD2 in this paper.

As stated above (section 3), we have previously shown that our FANCD2 ubiquitinated structures ($ID2_{Ub}$, $I_{Ub}D2_{Ub}$-DNA and USP1-UAF1-bound $ID2_{Ub}$-DNA) adopt the same conformation as the ones produced by FA-core complex and wild-type UBE2T/Ub. This is now stated in the second paragraph of the Results section in our revised manuscript.

1. ... Similarly, the biochemistry is done in the absence of the core complex which may change the kinetics of the ubiquitination and deubiquitination.
The remaining suggestions seems to be minor (repeat of biochemical experiment, …

Unfortunately, we have not established FA-core expression and purification in our lab to perform the relevant ubiquitination reactions. Doing so would require several months of work. Nevertheless, assays focused on FANCI ubiquitination have also shown the same result: "*…in agreement with what has been observed before with FA-core catalyzed reactions (Wang et al, 2021), we found that the rate of FANCI ubiquitination is significantly higher in $ID2_{Ub}$-DNA complex than in ID2-DNA complex (Fig. 3C)*." (second paragraph of Discussion). Similarly, we also expect that FANCD2 would be ubiquitinated significantly faster in $I_{Ub}D2$ than in ID2 complex, given also that the reaction step involving ID2 remodeling can be bypassed with $I_{Ub}D2$, since this complex is already in a closed-on-DNA state. Similarly, USP1-UAF1 catalyzed ID2 deubiquitination is also accelerated, when the closed ID2 state is compromised (Wang et al 2020, Nature). These arguments are now stated in a new paragraph in the discussion section, just after the mentioning of the interdependent ID2 ubiquitination mechanism (described in Figure 6).

Reviewer #3

USP1:UAF1 has been shown to have DNA binding activity (eg Liang et al 2016 Cell Reports). Could this DNA binding activity potentially reduce its activity on I-Ub-D2-Ub in the presence of DNA (ie through sequestration away from the substrate by binding to other DNA?). This could be tested by showing that titrating the amount of DNA in the reaction does not inhibit deubiquitination.

That is an interesting point. However, for DNA to be able to sequester UAF1 from ubiquitinated-ID2 complex, this would imply that USP1-UAF1-DNA binding is comparable or stronger to ID2-USP1-UAF1 binding. It is worth noting that UAF1 binding to DNA has been reported only via EMSA and not in solution, under physiological salt concentrations. We have previously shown that our PIFE DNA-binding assays are much more sensitive than EMSAs to NaCl concentration, and therefore much more likely to reflect true binding when physiological salt concentrations are used (Rennie et al 2020, EMBO reports). We have actually attempted to measure UAF1-DNA binding using PIFE assays, but found that there is virtually no affinity, in contrast with what has been observed with ID2 complexes. Additionally, no indications of UAF1-binding to DNA had been observed when assembling USP1-UAF1-$ID2_{Ub}$-dsDNA complex for cryo-EM analysis (Rennie et al 2020, Nature Structural and Molecular Biology): if there was competition for UAF1 binding, between ID2 and dsDNA, this would not favor the ternary complex formation which was by far the dominant well-resolved 3D class in that study. This indicates that even if there is some weak binding of USP1-UAF1 to dsDNA, both dsDNA and USP1-UAF1 preferentially bind to ubiquitinated ID2. Besides, if the mechanism of inhibition is due to USP1-UAF1 being

sequestered by DNA, this would not explain the differences in USP1-UAF1 resistance between ID2$_{Ub}$-DNA and ID2$_{Ub}$-DNA. For the above reasons we consider very unlikely that DNA can sequester USP1-UAF1 to provide deubiquitination protection to ID2. In response to the above, we have added the following sentences in the discussion section(4th paragraph): *"Although we cannot exclude the possibility of a UAF1-ssDNA binding event promoting ID2 deubiquitination, there is no evidence in support of dsDNA-UAF1 binding influencing ID2 deubiquitination: cryo-EM analysis of in vitro assembled USP1-UAF1-ID2$_{Ub}$-DNA complexes, suggests that formation of a ternary complex is favored, with both dsDNA and USP1-UAF1 preferentially binding ubiquitinated ID2, rather than each other (Rennie et al, 2021)."*

The most likely mechanism of dsDNA-dependent inhibition of USP1-UAF1 activity towards di-mono-ubiquitinated of FANCI-ubiquitinated ID2 complex is through stabilization of the closed ID2 conformation, as we already state in the discussion (5th paragraph in revised manuscript): *"The deubiquitination protection of I$_{Ub}$D2 and I$_{Ub}$D2$_{Ub}$ complexes by dsDNA is likely mediated through stabilization of the ubiquitinated ID2 complexes in the closed conformation in which FANCI's ubiquitin, and therefore FANCD2's ubiquitin too, are maximally protected. In support for this, the R1285Q mutant of FANCI, which is predicted to disrupt the closed-on-DNA conformation of ubiquitinated ID2 complexes (Wang et al, 2020; Rennie et al, 2020)(Fig. EV1), impairs both the dsDNA binding (Rennie et al, 2020) and the I$_{Ub}$/D2$_{Ub}$ protection from USP1-UAF1-mediated deubiquitination (Wang et al, 2020). While closed-conformation ubiquitinated ID2 complexes can exist in the absence of DNA, as previously shown for ID2$_{Ub}$ (Rennie et al, 2020), such DNA-free complexes may be less stable, or conformationally more flexible, without the avidity conferred by the interacting DNA. Therefore, they may be more amenable to deubiquitination. Our biochemical assays indicate that FANCI ubiquitination further secures this closed ID2 conformation. This is likely achieved through FANCI-ubiquitination effectively restricting the conformational inclination of the USP1-UAF1-bound ID2 complex towards the open-state conformation. Indeed, the structure of USP1-UAF1 complex bound to ID2$_{Ub}$-DNA revealed ID2 movements towards the open-state conformation, affecting not only FANCI helices in the region where ubiquitin-conjugation occurs, but also, and most profoundly, the FANCD2 N-terminus involved in interaction with FANCI's ubiquitin (Rennie et al, 2021)."*

1st Editorial Decision **8th Jul 2022**

Thank you again for submitting your revised Review Commons manuscript for consideration by The EMBO Journal. In light of the positive original comments and the interest of the subject of the study, we shall be happy to pursue a manuscript revised along the lines proposed in your co-submitted revision plan further for publication.

As mentioned before, my consultation with an expert in cryo-EM structure determination has raised some additional queries with regard to potential confounding effects of preferred orientation in the single-particle data, and the interpretability of side chain orientations/interactions in some of the figures. Based on our discussions of these issues, I would not expect new structural experiments at this point, but appreciate incorporation of further explanations and responses addressing these concerns in the final manuscript.

Dear Dr Vodermaier,

Thank you for inviting us to submit a revised manuscript of our work. We have now performed the additional experiments stated in our revision plan (DNA binding assays in the presence of excess ubiquitinated FANCD2, and FANCI deubiquitination assays in the presence of a FANCD2 mutant predicted to display impaired binding to FANCI's ubiquitin). Our new findings are now incorporated in Figures 2 and 5, respectively. We have additionally altered Fig EV2B and added an extra figure (EV4) in accordance with the recommendation to show cryo-EM maps at regions where side chain interactions were previously indicated. Lastly, we have also updated the text for minor errors and for addressing the issue of preferential orientation of particle images. In accordance to EMBO Journal guidelines, we have also trimmed our abstract to 173 words and have specified the type of replicates (technical/biological) used in each figure legend. We would like to thank you and the reviewers for helping us improve our manuscript. We hope that our revised version will be suitable for publication in EMBO Journal.

Please find below our detailed response to yours and the reviewers' comments.

Kind Regards

Kimon Lemonidis and Helen Walden

Editor/expert in cryo-EM

As mentioned before, my consultation with an expert in cryo-EM structure determination has raised some additional queries with regard to potential confounding effects of preferred orientation in the single-particle data, and the interpretability of side chain orientations/interactions in some of the figures. Based on our discussions of these issues, I would not expect new structural experiments at this point, but appreciate incorporation of further explanations and responses addressing these concerns in the final manuscript.

The preferred orientation we encountered does not seem to be unique for our specific ID2 complex, and may be a common feature for ID2 heterodimers, as a number of ID2 complexes (non-ubiquitinated or FANCD2 ubiquitinated) have been shown to also exhibit preferential orientation of particle images. The preferred orientation of our particle images did not prevent us from obtaining a good structural model (with no rotamer/Ramachadran outliers), displaying regions of adequately high resolution. It is worth noting that our model was produced used a higher resolution template which has essentially the same conformation with our structure. Overall, our map and model is of sufficient resolution to support our main conclusions: 1) I_{Ub}D2 complex is in a closed-on-DNA conformation, 2) K561 of FANCD2 is exposed for ubiquitination, and 3) The ubiquitin conjugated on FANCI has an extended interface with FANCD2, which is also supported by the $I_{Ub}D2_{Ub}$ structure and our new biochemical experiments with the $D2^{H209A,\ VP243AA}$ mutant (Figure 5).

We have thus added the following sentences in our results section: "*The presence of such smaller particles, combined with I_{Ub}D2-DNA-specific particles adopting a preferred orientation (Fig. EV1D), restricted to some extent the overall resolution of our final map. Similarly, chicken ID2 and $ID2_{Ub}$ complexes (containing either wild-type or phosphomimetic FANCI) have been reported to exhibit too, a preferred orientation of cryo-EM particles images (Alcón et al, 2020; Sijacki et al, 2022). Hence, a strikingly uneven angular distribution of cryo-EM particle images may be a common feature shared by ID2 complexes, irrespective of ubiquitination.*"… "Despite the limited overall map resolution, our maps had several well-resolved regions for modelling..."

Regarding the interpretability of side chains, we have previously displayed side chains of our I_{Ub}D2-DNA structure in three parts in our manuscript:

1) Fig. 3B, upon recommendation of reviewer #1 to include measurement of target lysines to the NTD of the other subunit. Here, we would argue that the exact orientation of the two side chains shown is less relevant, as these just serve as reference for the distance, which is evidently greater than in the ID2 complex (also evident in Fig EV3).

2) Fig. 5A. For these side chains and associated interactions we now have a figure (Fig EV4) illustrating how these residues fit to our cryo-EM map. While our map resolution is variable in this region, with certain side-chains better resolved than others, the overall conformation/structure of our complex is nearly identical to the one of the higher resolution $I_{Ub}D2_{Ub}$-DNA structure (Fig. 5A), with associated interactions highly conserved, suggesting high confidence in the orientation of illustrated side chains.

3) Fig EV2B. Our map resolution is rather poor at this region. We thus decided to only show there how helices in that region are positioned slightly differently in I_{Ub}D2 and $I_{Ub}D2_{Ub}$ structures, by displaying the respective aligned structures (main chain only) along with our cryo-EM map.

Reviewer #1

1. The major caveat is that these structures were generated using "engineered Ube2T and SpyCatcher-SpyTag", and not via the FA core complex. Recent structural work by the Passmore and Pavletich labs shows the intricate process of ID2 complex ubiquitination, and propose a sequential ubiquitination model that is tied closely to the unique structural properties of the FA core and the ID2 interface. Similarly, the biochemistry is done in the absence of the core complex which may change the kinetics of the ubiquitination and deubiquitination. So, although the biochemical experiments performed are robust, these caveats need to be explicitly noted in the manuscript.

We agree that the kinetics of ubiquitination may be somewhat different when an engineered UBE2T is used along with a truncated FANCL, as opposed to wild type UBE2T and FA-core complex. However, we have previously shown that our approach results in site specific modification of FANCD2 and FANCI (Chaugule et al 2019 Methods in Enzymology; Chaugule et al 2020 Nature Chemical Biology) and in ID2 complexes having the same closed-on-DNA conformation (Rennie et al 2020 EMBO reports; Rennie et al 2021; Nature Structural and Molecular Biology) as the one observed with complexes produced via wild-type UBE2T and FA-core complex (Alcon et al 2020 Nature Structural and Molecular Biology; Wang et al 2020 Nature). This suggests that ID2 ubiquitination is actually responsible for both producing and maintaining the final ID2 conformation. Hence the conformation of our complex would also be determined by its ubiquitinated status. Accordingly, our deubiqutination assays would be unaffected by the way our ubiquitinated complexes were formed, since these are structurally indistinguishable from complexes produced using FA-core catalyzed reactions. Regarding the biochemistry experiments, we should note that wild-type tag-less ubiquitin was used in our *in vitro* ubiquitination reactions, whereas the tag on ubiquitin (GST-spyCatcher-Spytag or GST-tag) had been removed during preparation of ubiquitinated FANCI/FANCD2, and hence our deubiqutination assays also occurred on tag-less ubiquitin. Lastly, irrespective of any differences in kinetics between the two ubiquitination approaches, our results are in agreement with what had been observed also with FA-core: FANCI ubiquitination progresses at a much faster rate once FANCD2 is ubiquitinated. Although we have no FA-core catalyzed control for I_{Ub}D2 vs ID2 ubiquitination, we do not see any reason why FANCD2 would not be ubiquitinated faster in I_{Ub}D2 complex in that case: FA-core has been shown to ubiquitinate FANCD2 by altering the conformation of the ID2 complex to a closed-on-DNA state to achieve catalysis. In I_{Ub}D2 complex this step can be

bypassed, since this complex is already in the closed conformation, and hence the reaction is expected to progress faster.

To reflect the above, we have modified the part where we describe how we created the I_{Ub}D2-DNA complex (in Results section) into: "*To address these questions, we used our in vitro reconstitution approach (Arkinson et al, 2018; Rennie et al, 2020, 2021) to assemble a 1:1:1 I_{Ub}D2-DNA complex, from purified I_{Ub}, D2 and dsDNA (61 bp long), and subsequently determined its structure by cryo-EM. Such reconstitution approach has been successfully applied to produce ID2_{Ub}/I_{Ub}D2_{Ub}-DNA structures (Rennie et al, 2020) and USP1-UAF1-bound ID2_{Ub}-DNA structures (Rennie et al, 2021). These complexes have been shown to adopt the same closed ID2 conformation as the one observed in ubiquitinated ID2-DNA complexes produced following FA-core-catalyzed ID2 ubiquitination (Alcón et al, 2020; Wang et al, 2020). This suggests that, despite the requirement of FA-core for opening up the ID2 complex for subsequent ubiquitination (Wang et al, 2021), ID2 ubiquitination is actually required for both producing and maintaining the final closed ID2 conformation. Hence, we reasoned that our in vitro assembled complex would also be structurally indistinguishable from a complex produced through removal of FANCD2's ubiquitin from I_{Ub}D2_{Ub}.*"

We have also included an additional paragraph in discussion in support of our findings shown in Fig. 3C-D, quoting agreements with previous *in vitro* assays using FA-core, as well as with the mechanism of FA-core action (Wang et al 2021, Nature Structural and Molecular Biology). In this paragraph, we also give an analogy whereby USP1-UAF1 catalyzed ID2 deubiquitination is also accelerated, when the closed ID2 state is compromised (Wang et al 2020, Nature)

2. Ideally, the authors should also solve cryo EM structure of IUbD2Ub and compare this to 6VAE to show that their in vitro ID2 assembly leads to structures aligning with the FA-core complex ubiquitinated form of ID2.

Solving the cryo EM structure of IUbD2Ub is not a trivial ask, but the authors may already have it or may be inclined to do it since they already have access to purified IUb D2Ub and have optimized cryo EM conditions for IUbD2 in this paper.

As stated above, we have previously shown that our FANCD2 ubiquitinated structures (ID2_{Ub}, I_{Ub}D2_{Ub}-DNA and USP1-UAF1-bound ID2_{Ub}-DNA) adopt the same conformation as the ones produced by FA-core complex and wild-type UBE2T/Ub. This is now stated in the second paragraph of the Results section in our revised manuscript.

3. I would suggest including ubiquitinated FANCD2 in the PIFE experiments in Figure 2, to assess differential binding affinity of I and IUb. This would help serve as a control that FANCD2 ubiquitination also strengthens the ID2 complex binding the DNA.

We have now performed PIFE assays also assessing the affinity of I and I_{Ub} to DNA in the presence of excess ubiquitinated FANCD2. We found that all alternatively ubiquitinated ID2 complexes (I_{Ub}D2, ID2_{Ub}, I_{Ub}D2_{Ub}), display comparable affinities to dsDNA, which are considerably higher (>10 fold) than the one observed for non-ubiquitinated ID2 complex. Figure 2B and associated text in results, methods and figure legend have been altered accordingly to incorporate these new data. Due to some variations of amplitude response (difference in fluorescence between unbound and bound states) among various experiments, we have now normalised all baseline-subtracted PIFE values against the observed amplitude, assuming unbound and bound values of 0 and 1, respectively, for each of the protein/complex assessed. The Kd values previously estimated for I_{Ub}D2, ID2 and D2 are in agreement with the ones produced after normalisation.

4. The authors claim that "ubiquitination of either of the two subunits of ID2 (FANCI or FANCD2) actually favors ubiquitination of the other subunit" (supported by biochemical and structural data in Figure 3). In Figure 3A-B, it is difficult to draw a conclusion on the "accessibility of the lysine residues" from these figures. Authors should include a measurement of the distances between the Lysines and the NTD of the opposite subunit. This would add a numeric measure to "accessibility".

We have now modified panel B of Figure 3 to better illustrate how FANCI/FANCD2 lysine accessibility improves when the other subunit is ubiquitinated. We specifically show for each of the two lysines (epsilon amino group), all the residues (side-chains) of the other subunit located within 8 Å, and how the distance to the closest residue increases upon ubiquitination of the other subunit. We have additionally incorporated a table (Fig. EV3B) displaying accessible/buried surface areas for these lysines for all available ID2 complexes.

5. Wang et al, 2020 show that the FANCI K523 is only accessible for ubiquitination upon prior FANCD2 ubiquitination. However, the IUbD2 state could be formed by FANCD2-deubiquitination of IUbD2Ub. Figure 5 may need to changed to remove the arrow ID2 state to IUbD2.

We have now removed this arrow in that Figure (which is now Figure 6 in the manuscript).

6. In Figure 3B, the structure of IUbD2Ub should be included to allow a proper comparison of the Lysine accessibility of IUbD2 and ID2Ub versus IUbD2Ub.

We have now incorporated in Fig 3B for comparison, images of $I_{Ub}D2_{Ub}$ aligned with the FANCD2 of $I_{Ub}D2$ (left panel), or with FANCI of $ID2_{Ub}$ (right panel).

7. The final paragraph needs to be reworded. The following sentence is very confusing "Of these two interfaces, one is absent in equivalent ubiquitin-FANCI interactions in ID2Ub-DNA structure (Wang et al, 2020)(Fig. 4D-E)." It does not come across that ubiquitin of FANCIUb lacks an interface with FANCD2.

We agree that there may be some confusion relating to the identity of ubiquitin and corresponding complex when these interfaces are mentioned and compared. We have thus modified this paragraph to: *"*Whereas the FANCI interaction with the ubiquitin conjugated to FANCD2 has no protective role against USP1-UAF1 mediated FANCD2 deubiquitination, the interaction of FANCI's ubiquitin with FANCD2 (in $I_{Ub}D2$-DNA and $I_{Ub}D2_{Ub}$-DNA complexes) efficiently protects against FANCI deubiquitination. In each case, ubiquitin's hydrophobic I44 patch is involved in interaction with the other ID2 subunit; however, the ubiquitin conjugated to FANCI forms a more extended interface with FANCD2 (Wang *et al*, 2020)(Fig. 5A). This extended interface is formed predominantly via hydrophobic interactions of residues H209 (α10 helix), V243, P244 and D247 (α13 helix) of FANCD2 with residues T9 and K11 of ubiquitin, and is further stabilized by hydrogen bonding between ubiquitin's T9 and R74 with FANCD2's H209 and S251 (or E217), respectively (Fig. 5A). The residues of FANCD2 predicted to be involved in this extended interface…*"*

Minor

1. Please include specific method of expressing, purifying and ubiquitinating FANCI and FANCD2. This method doesn't become clear by referencing previous papers, and these methods might have changed.

We have now given a brief description of the methods employed for producing ubiquitinated and non-ubiquitinated versions of FANCI/FANCD2 and related ID2 complexes, and also of methods employed for producing USP1, USP1ΔN, UAF1 and related complexes.

2. "Our structure indicates that this ubiquitin-FANCD2 interface ..." probably should be "Our structure indicates that the interface between FANCD2 and the ubiquitin of FANCIUb ..."

We have reworded the whole paragraph as mentioned above. This is now changed into: *"This extended interface is formed predominantly via hydrophobic interactions of residues …"*

3. I would suggest adding additional supplemental figures of Figure 3B including 1 extra angle for each structure. This would help gauge "Lysine accessibility".

Figure 3B now consists of focused images of FANCD2-K561 and FANCI-K523 region, showing their accessibility in ID2, $I_{Ub}D2$, $ID2_{Ub}$ and $I_{Ub}D2_{Ub}$ structures (with residues of the other subunit within 8Å and measurements of lysine-NTD distances shown). The comparison of entire ID2, $I_{Ub}D2$, $ID2_{Ub}$ and $I_{Ub}D2_{Ub}$ structures shown before, is now moved to Fig. EV3A, with extra numerical details of lysine accessibility shown in Fig. EV3B.

4. Please add PDB images in Figure 4D from the IUbD2Ub structure of both the interface between FANCD2 and ubiquitin of FANCIUb, and FANCI and the ubiquitin of FANCD2Ub. This is important to allow for the comparison of the different ubiquitin interfaces amongst the IUbD2Ub, ID2Ub, and IUbD2 structures

We have now included illustrations of respective ubiquitin-FANCI and ubiquitin-FANCD2 interactions in $I_{Ub}D2_{Ub}$ structure for direct comparison. Due to the increased length of information shown (with results from extra experiments to be also included in the final revision), these, along with the amino-acid conservation subfigure, are now illustrated in a separate figure (Figure 5).

Reviewer #2

Some mutagenesis studies that investigate the hypotheses generated by the structure would increase the overall impact of the study.

We have now produced a FANCD2 H209A, VP243AA mutant and assessed how this mutation impacts on FANCI deubiquitination. Such mutation is predicted to disrupt the extended interface between FANCD2 and the ubiquitin conjugated on FANCI. We found that the FANCD2 H209A/VP243AA mutant provides less resistance to FANCI deubiquitination, compared to wild-type FANCD2 (Fig 5C). We have additionally assessed FANCI deubiquitination at increasing concentrations of FANCD2 (wild-type or H209A/VP243AA mutant) and found that when FANCD2 is excess, FANCI is deubiquitinated more robustly when associated with the H209A/VP243AA mutant, rather than with the wild-type FANCD2 (Fig 5D). The above results indicate that the extended interface between FANCD2 and the ubiquitin on FANCI is required for enhanced FANCI protection against deubiquitination. Figure 5, and corresponding text in figure legend, results, materials and methods and discussion sections have been altered appropriately to incorporate these findings.

The major limitions of this study is the lack of functional connection to the described mechanisms of deubiquitylation or reubiquitylation of FANCI-FANCD2 complexes. In the future, hopefully, this group or others will generate point mutants

that can selectively separate the functions of FANCI or FANCD2 deubiquitylation or FANCI or FANCD2 reubiquitylation to show the importance of dynamic regulation of these processes in a cellular or in vivo context

The scope of this work was in providing a biochemical and structural explanation of the interdependency in FANCI-FANCD2 ubiquitination previously observed *in vivo*. Providing a functional connection of some of our *in vitro* and structural findings, however, is a long term aim in our group. Nevertheless, we believe that important aspects relating to the mechanism of FANCI ubiquitination and deubiquitination need to be uncovered first to allow generation of mutants that selectively affect ubiquitination and deubiquitination of each of the two ID2 subunits. To address the importance of unravelling the mechanism of FANCI ubiquitination and deubiquitination for future *in vivo* studies, we have a created a separate paragraph highlighting this in the discussion.

A major comment is whether the authors can by structural inference figure out what part of USP1 N-terminal extension is required for interaction and recognition of FANCI-FANCD2-Ub-DNA complexes. This is the only part of the study that wasn't very satisfying. Perhaps the authors can address this with some modeling and/or with additional mutational analyses on USP1.

We have previously shown that residues R22 and L23 of USP1 N-terminus are involved in FANCD2 binding (Arkinson, et al 2018, Life Science Alliance), which is supported by recently AlphaFold simulations performed in our group (Rennie et al 2022; bioRxiv). These are now stated in the Results section, just after the sentence: *"The above suggest that the interaction of FANCD2's ubiquitin with FANCI (in the* $I_{Ub}D2_{Ub}$*-DNA complex) does not protect against FANCD2 deubiquitination, as the latter can be efficiently achieved by a mechanism that involves USP1's N-terminus".*

One minor comment is that the authors only cite the Smorgorzewska et al, Cell, 2007 paper for observing the first interdependency of FANCI-FANCD2 ubiquitylation in cells (Citations in the Intro and Discussion sections). The authors should also cite Sims et al NSMB, 2007 for the exact same observation shown in the two papers just to be consistent.

The reason that this article was not referenced in instances where this interdependency was mentioned, was because that study did not show that FANCD2 ubiquitination is dependent on FANCI ubiquitination, but rather that loss of FANCI (or certain FANCI mutations) disrupts FANCD2 ubiquitination. Regardless, we agree that this study should be given credit and be cited along with the Smorgorzewska et al, Cell, 2007 paper, for showing that FANCI ubiquitination depends on FANCD2 ubiquitination and for providing evidence which are consistent with the interdependency observed by Smorgorzewska et al. We have now included this reference in parts where interdependent FANCI-FANCD2 ubiquitination is mentioned.

Reviewer #3

Minor comments: The role of FANCI-Ub and DNA binding in protecting both FANCI and FANCD2 from deubiquitination was first shown in van Twest et al 2017 Molecular Cell, however this is not referred to in the manuscript.

To incorporate this, we have now altered the part where the protective role of DNA is mentioned in the results section, into: *"Previous work has shown that DNA is required for efficient protection of both FANCI and FANCD2 against USP1-UAF1 mediated deubiquitination (van Twest et al, 2017; Arkinson et al, 2018). Focusing on FANCD2 deubiquitination, we have previously found that FANCI ubiquitination (but not FANCI itself) is additionally required for restricting FANCD2 deubiquitination, but has no effect in protecting from FANCD2 deubiquitination, in the absence of dsDNA (Arkinson et al, 2018)."*

Also, one previous study showed that DNA *stimulates* deubiquitination of FANCD2. This study used complexes where FANCI was not ubiquitinated, but is a different result to what is seen in Figure 4A (but perhaps because deubiquitination is already 100% in the absence of DNA in these experiments). Some investigation/discussion of this discrepancy would be good to include. USP1:UAF1 has been shown to have DNA binding activity (eg Liang et al 2016 Cell Reports). Could this DNA binding activity potentially reduce its activity on I-Ub-D2-Ub in the presence of DNA (ie through sequestration away from the substrate by binding to other DNA?). This could be tested by showing that titrating the amount of DNA in the reaction does not inhibit deubiquitination.

Although we cannot exclude the possibility that such discrepancies may derive from differences in the way the respective protein/complexes were produced, the main difference between our assays and theirs, is in the type of DNA used (ssDNA vs dsDNA). It is thus likely that ubiquitinated ID2 complexes produced by a DNA structure other than dsDNA may be more amenable to deubiquitination by USP1-UAF1. Otherwise our results with dsDNA are very reproducible and also in agreement with what has been reported by another group (van Twest et al, 2017). We have thus changed DNA into dsDNA in several parts of the discussion to distinguish it from ssDNA (or other DNA structures) and modified the discussion a bit to also include the following sentences regarding the work of Liang et al 2016, 2019 (4th paragraph): *"Interestingly, the opposite effect (DNA promoting USP1-UAF1-mediated FANCD2 deubiquitination) has been reported in a study utilizing a ~60% FANCD2-ubiquitinated ID2 complex produced with the aid of a 64-mer single-stranded DNA (Liang et al, 2019). While the reasons for such discrepancy need to be further investigated, it is likely that the source of DNA (singe- versus double- stranded) used for ID2 ubiquitination, determines whether FANCD2 deubiquitination will be promoted or inhibited. UAF1 has been shown to bind both double-stranded (dsDNA), single-stranded (ssDNA), or more complex D-loop structures of DNA, in vitro (Liang et al, 2016, 2019). Although we cannot exclude the possibility of a UAF1-ssDNA binding event promoting ID2 deubiquitination, there is no evidence in support of dsDNA-UAF1 binding influencing ID2 deubiquitination: cryo-EM analysis of in vitro assembled USP1-UAF1-ID2$_{Ub}$-DNA complexes, suggests that formation of a ternary complex is favored, with both dsDNA and USP1-UAF1 preferentially binding ubiquitinated ID2, rather than each other (Rennie et al, 2021)."*

The idea of DNA inhibiting ID2 deubiquitination by sequestering UAF1 is interesting. However, for DNA to be able to sequester UAF1 from ubiquitinated-ID2 complex, this would imply that USP1-UAF1-DNA binding is comparable or stronger to ID2-USP1-UAF1 binding. It is worth noting that UAF1 binding to DNA has been reported only via EMSA and not in solution, under physiological salt concentrations. We have previously shown that our PIFE DNA-binding assays are much more sensitive than EMSAs to NaCl concentration, and therefore much more likely to reflect true binding when physiological salt concentrations are used (Rennie et al 2020, EMBO reports). We have actually attempted to measure UAF1-DNA binding using PIFE assays, but found that there is virtually no affinity, in contrast with what has been observed with ID2 complexes. Additionally, no indications of UAF1-binding to DNA had been observed when assembling USP1-UAF1-ID2$_{Ub}$-dsDNA complex for cryo-EM analysis (Rennie et al 2020, Nature Structural and Molecular Biology): if there was competition for UAF1 binding, between ID2 and dsDNA, this would not favor the ternary complex formation which was by far the dominant well-resolved 3D class in that study. This indicates that even if there is some weak binding of USP1-UAF1 to dsDNA, both dsDNA and USP1-UAF1 preferentially bind to ubiquitinated ID2. Besides, if the mechanism of inhibition is due to USP1-UAF1 being sequestered by DNA, this would not explain the differences in USP1-UAF1 resistance between ID2$_{Ub}$-DNA and ID2$_{Ub}$-DNA. For the above reasons we consider very unlikely that DNA can sequester USP1-UAF1 to provide deubiquitination protection to ID2. In response to the above, we have added the following sentences in the discussion section(4th paragraph): *"Although we cannot exclude the*

possibility of a UAF1-ssDNA binding event promoting ID2 deubiquitination, there is no evidence in support of dsDNA-UAF1 binding influencing ID2 deubiquitination: cryo-EM analysis of in vitro assembled USP1-UAF1-ID2$_{Ub}$-DNA complexes, suggests that formation of a ternary complex is favored, with both dsDNA and USP1-UAF1 preferentially binding ubiquitinated ID2, rather than each other (Rennie et al, 2021)."

The most likely mechanism of dsDNA-dependent inhibition of USP1-UAF1 activity towards di-mono-ubiquitinated of FANCI-ubiquitinated ID2 complex is through stabilization of the closed ID2 conformation, as we already state in the discussion (5th paragraph in revised manuscript): *"The deubiquitination protection of I$_{Ub}$D2 and I$_{Ub}$D2$_{Ub}$ complexes by dsDNA is likely mediated through stabilization of the ubiquitinated ID2 complexes in the closed conformation in which FANCI's ubiquitin, and therefore FANCD2's ubiquitin too, are maximally protected. In support for this, the R1285Q mutant of FANCI, which is predicted to disrupt the closed-on-DNA conformation of ubiquitinated ID2 complexes (Wang et al, 2020; Rennie et al, 2020)(Fig. EV1), impairs both the dsDNA binding (Rennie et al, 2020) and the I$_{Ub}$/D2$_{Ub}$ protection from USP1-UAF1-mediated deubiquitination (Wang et al, 2020). While closed-conformation ubiquitinated ID2 complexes can exist in the absence of DNA, as previously shown for ID2$_{Ub}$ (Rennie et al, 2020), such DNA-free complexes may be less stable, or conformationally more flexible, without the avidity conferred by the interacting DNA. Therefore, they may be more amenable to deubiquitination. Our biochemical assays indicate that FANCI ubiquitination further secures this closed ID2 conformation. This is likely achieved through FANCI-ubiquitination effectively restricting the conformational inclination of the USP1-UAF1-bound ID2 complex towards the open-state conformation. Indeed, the structure of USP1-UAF1 complex bound to ID2$_{Ub}$-DNA revealed ID2 movements towards the open-state conformation, affecting not only FANCI helices in the region where ubiquitin-conjugation occurs, but also, and most profoundly, the FANCD2 N-terminus involved in interaction with FANCI's ubiquitin (Rennie et al, 2021)."*

Some further discussions speculating why a FANCI-Ub is necessary in the FA pathway (ie why not just have FANCD2-Ub as the clamping mechanism given that it is sufficient to lock the protein onto DNA?) would increase the interpretation of the work by non-specialists.

Our work shows that FANCI ubiquitination is required for maintaining the ID2 clamping on DNA, which would otherwise be rapidly lost through deubiquitination of FANCD2. To elaborate this we have added to the Discussion (end of second paragraph) the following sentences: *"…, and therefore ubiquitinated ID2 cannot easily revert to a non-ubiquitinated state. In essence, FANCI ubiquitination, via maintaining FANCD2 ubiquitination, commits the ID2$_{Ub}$ complex for FA repair, since without FANCI ubiquitination such complex would be rapidly transformed to a non-ubiquitinated ID2 complex, through the action of USP1-UAF1."*

The work has clinical relevance - USP1:UAF1 inhibitors and FA core complex inhibitors are being developed by several biotech for cancer therapy (this is not referenced in the manuscript, and could perhaps be included in the introduction?).

We have now included text and references highlighting the clinical relevance of this study in the penultimate paragraph of our introduction. We thank the reviewer for pointing this out.

1st Revision - Editorial Decision 25th Oct 2022

Thank you for submitting your revised manuscript for our consideration. I am pleased to inform you that in light of the positive re-review by one of the original Review Commons referees (see below), we have now accepted it for publication in The EMBO Journal!

Referee #1:

I am satisfied with the authors responses to my comments and suggestions. The manuscript is a nice complement to the recently-published work on the Ub of the ID complex.

EMBO Press Author Checklist

Corresponding Author Name: Kimon Lemonidis	
Journal Submitted to: EMBO Journal	
Manuscript Number: EMBOJ-2022-111898	

USEFUL LINKS FOR COMPLETING THIS FORM
[The EMBO Journal - Author Guidelines]
[EMBO Reports - Author Guidelines]
[Molecular Systems Biology - Author Guidelines]
[EMBO Molecular Medicine - Author Guidelines]

Reporting Checklist for Life Science Articles (updated January
This checklist is adapted from Materials Design Analysis Reporting (MDAR) Checklist for Authors. MDAR establishes a minimum set of requirements in transparent reporting in the life sciences (see Statement of Task: 10.31222/osf.io/9sm4x). Please follow the journal's guidelines in preparing your
Please note that a copy of this checklist will be published alongside your article.

Abridged guidelines for figures
1. Data
The data shown in figures should satisfy the following conditions:
- the data were obtained and processed according to the field's best practice and are presented to reflect the results of the experiments in an accurate and unbiased manner.
- ideally, figure panels should include only measurements that are directly comparable to each other and obtained with the same assay.
- plots include clearly labeled error bars for independent experiments and sample sizes. Unless justified, error bars should not be shown for technical
- if n<5, the individual data points from each experiment should be plotted. Any statistical test employed should be justified.
- Source Data should be included to report the data underlying figures according to the guidelines set out in the authorship guidelines on Data

2. Captions
Each figure caption should contain the following information, for each panel where they are relevant:
- a specification of the experimental system investigated (eg cell line, species name).
- the assay(s) and method(s) used to carry out the reported observations and measurements.
- an explicit mention of the biological and chemical entity(ies) that are being measured.
- an explicit mention of the biological and chemical entity(ies) that are altered/varied/perturbed in a controlled manner.
- the exact sample size (n) for each experimental group/condition, given as a number, not a range;
- a description of the sample collection allowing the reader to understand whether the samples represent technical or biological replicates (including how many animals, litters, cultures, etc.).
- a statement of how many times the experiment shown was independently replicated in the laboratory.
- definitions of statistical methods and measures:
  - common tests, such as t-test (please specify whether paired vs. unpaired), simple χ2 tests, Wilcoxon and Mann-Whitney tests, can be unambiguously identified by name only, but more complex techniques should be described in the methods section;
  - are tests one-sided or two-sided?
  - are there adjustments for multiple comparisons?
  - exact statistical test results, e.g., P values = x but not P values < x;
  - definition of 'center values' as median or average;
  - definition of error bars as s.d. or s.e.m.

Please complete ALL of the questions below.
Select "Not Applicable" only when the requested information is not relevant for your study.

Materials

Newly Created Materials	Information included in the manuscript?	In which section is the information available? (Reagents and Tools Table, Materials and Methods, Figures, Data Availability Section)
New materials and reagents need to be available; do any restrictions apply?	Not Applicable	

Antibodies	Information included in the manuscript?	In which section is the information available? (Reagents and Tools Table, Materials and Methods, Figures, Data Availability Section)
For **antibodies** provide the following information:<br>- Commercial antibodies: RRID (if possible) or supplier name, catalogue number and or/clone number<br>- Non-commercial: RRID or citation	Yes	Materials and Methods

DNA and RNA sequences	Information included in the manuscript?	In which section is the information available? (Reagents and Tools Table, Materials and Methods, Figures, Data Availability Section)
**Short novel DNA or RNA including primers, probes**: provide the sequences.	Yes	Materials and Methods

Cell materials	Information included in the manuscript?	In which section is the information available? (Reagents and Tools Table, Materials and Methods, Figures, Data Availability Section)
**Cell lines:** Provide species information, strain. Provide accession number in repository **OR** supplier name, catalog number, clone number, and/**OR** RRID.	Not Applicable	
**Primary cultures:** Provide species, strain, sex of origin, genetic modification status.	Not Applicable	
Report if the cell lines were recently **authenticated** (e.g., by STR profiling) and tested for mycoplasma contamination.	Not Applicable	

Experimental animals	Information included in the manuscript?	In which section is the information available? (Reagents and Tools Table, Materials and Methods, Figures, Data Availability Section)
**Laboratory animals or Model organisms:** Provide species, strain, sex, age, genetic modification status. Provide accession number in repository **OR** supplier name, catalog number, clone number, **OR** RRID.	Not Applicable	
**Animal observed in or captured from the field:** Provide species, sex, and age where possible**.**	Not Applicable	
Please detail **housing and husbandry conditions**.	Not Applicable	

Plants and microbes	Information included in the manuscript?	In which section is the information available? (Reagents and Tools Table, Materials and Methods, Figures, Data Availability Section)
**Plants:** provide species and strain, ecotype and cultivar where relevant, unique accession number if available, and source (including location for collected wild specimens).	Not Applicable	
**Microbes:** provide species and strain, unique accession number if available, and source.	Not Applicable	

Human research participants	Information included in the manuscript?	In which section is the information available? (Reagents and Tools Table, Materials and Methods, Figures, Data Availability Section)
If collected and within the bounds of privacy constraints report on age, sex and gender or ethnicity for all study participants.	Not Applicable	

Core facilities	Information included in the manuscript?	In which section is the information available? (Reagents and Tools Table, Materials and Methods, Figures, Data Availability Section)
If your work benefited from core facilities, was their service mentioned in the acknowledgments section?	Yes	Materials and Methods

Design

Study protocol	Information included in the manuscript?	In which section is the information available? (Reagents and Tools Table, Materials and Methods, Figures, Data Availability Section)
If study protocol has been **pre-registered, provide DOI in the manuscript**. For clinical trials, provide the trial registration number **OR** cite DOI.	Not Applicable	
Report the **clinical trial registration number** (at ClinicalTrials.gov or equivalent), where applicable.	Not Applicable	

Laboratory protocol	Information included in the manuscript?	In which section is the information available? (Reagents and Tools Table, Materials and Methods, Figures, Data Availability Section)
Provide DOI OR other citation details if **external detailed step-by-step protocols** are available.	Yes	Materials and Methods

Experimental study design and statistics	Information included in the manuscript?	In which section is the information available? (Reagents and Tools Table, Materials and Methods, Figures, Data Availability Section)
Include a statement about **sample size** estimate even if no statistical methods were used.	Yes	Figure legends
Were any steps taken to minimize the effects of subjective bias when allocating animals/samples to treatment (e.g. **randomization procedure**)? If yes, have they been described?	Not Applicable	
Include a statement about **blinding** even if no blinding was done.	Not Applicable	
Describe **inclusion/exclusion criteria** if samples or animals were excluded from the analysis. Were the criteria pre-established?<br><br>If sample or data points were omitted from analysis, report if this was due to attrition or intentional exclusion and provide justification.	Yes	Materials and Methods
For every figure, are **statistical tests** justified as appropriate? Do the data meet the assumptions of the tests (e.g., normal distribution)? Describe any methods used to assess it. Is there an estimate of variation within each group of data? Is the variance similar between the groups that are being statistically compared?	Yes	Materials and Methods

Sample definition and in-laboratory replication	Information included in the manuscript?	In which section is the information available? (Reagents and Tools Table, Materials and Methods, Figures, Data Availability Section)
In the figure legends: state number of times the experiment was **replicated** in laboratory.	Yes	Figure legends
In the figure legends: define whether data describe **technical or biological replicates**.	Yes	Figure legends

Ethics

Ethics	Information included in the manuscript?	In which section is the information available? (Reagents and Tools Table, Materials and Methods, Figures, Data Availability Section)
Studies involving **human participants**: State details of **authority granting ethics approval** (IRB or equivalent committee(s), provide reference number for approval.	Not Applicable	
Studies involving **human participants**: Include a statement confirming that **informed consent** was obtained from all subjects and that the experiments conformed to the principles set out in the WMA Declaration of Helsinki and the Department of Health and Human Services Belmont Report.	Not Applicable	
Studies involving **human participants:** For publication of **patient photos**, include a statement confirming that consent to publish was obtained.	Not Applicable	
Studies involving experimental **animals**: State details of **authority granting ethics approva**l (IRB or equivalent committee(s), provide reference number for approval. Include a statement of compliance with ethical regulations.	Not Applicable	
Studies involving **specimen and field samples:** State if relevant **permits** obtained, provide details of authority approving study; if none were required, explain why.	Not Applicable	

Dual Use Research of Concern (DURC)	Information included in the manuscript?	In which section is the information available? (Reagents and Tools Table, Materials and Methods, Figures, Data Availability Section)
Could your study fall under dual use research restrictions? Please check biosecurity documents and list of **select agents and toxins** (CDC): https://www.selectagents.gov/sat/list.htm	Not Applicable	
If you used a select agent, is the security level of the lab appropriate and reported in the manuscript?	Not Applicable	
If a study is subject to dual use research of concern regulations, is the name of the **authority granting approval and reference number** for the regulatory approval provided in the manuscript?	Not Applicable	

Reporting

The MDAR framework recommends adoption of discipline-specific guidelines, established and endorsed through community initiatives. Journals have their own policy about requiring specific guidelines and recommendations to complement MDAR.

Adherence to community standards	Information included in the manuscript?	In which section is the information available? (Reagents and Tools Table, Materials and Methods, Figures, Data Availability Section)
State if relevant guidelines or checklists (e.g., **ICMJE, MIBBI, ARRIVE, PRISMA**) have been followed or provided.	Not Applicable	
For **tumor marker prognostic studies,** we recommend that you follow the **REMARK** reporting guidelines (see link list at top right). See author guidelines, under 'Reporting Guidelines'. Please confirm you have followed these guidelines.	Not Applicable	
For **phase II and III randomized controlled trials**, please refer to the **CONSORT** flow diagram (see link list at top right) and submit the CONSORT checklist (see link list at top right) with your submission. See author guidelines, under 'Reporting Guidelines'. Please confirm you have submitted this list.	Not Applicable	

Data Availability

Data availability	Information included in the manuscript?	In which section is the information available? (Reagents and Tools Table, Materials and Methods, Figures, Data Availability Section)
Have **primary datasets** been deposited according to the journal's guidelines (see 'Data Deposition' section) and the respective accession numbers provided in the Data Availability Section?	Yes	Data Availability Section
Were **human clinical and genomic datasets** deposited in a public access-controlled repository in accordance to ethical obligations to the patients and to the applicable consent agreement?	Not Applicable	
Are **computational models** that are central and integral to a study available without restrictions in a machine-readable form? Were the relevant accession numbers or links provided?	Not Applicable	
If publicly available data were reused, provide the respective **data citations in the reference list**.	Not Applicable	
